# Maternal Liver Retinol Stores Do Not Ensure Adequate Calf Vitamin A Under Restricted Late‐Gestation Vitamin A Intake

**DOI:** 10.1111/asj.70175

**Published:** 2026-04-10

**Authors:** Hannah F. Speer, Harvey C. Freetly, Mary E. Drewnoski

**Affiliations:** ^1^ University of Nebraska‐Lincoln Lincoln Nebraska USA; ^2^ USDA, ARS, US Meat Animal Research Center, Clay Center Nebraska USA

**Keywords:** beef cows, calves, liver, maternal nutrition, retinol, vitamin A

## Abstract

Vitamin A is critical for immune function and epithelial integrity, yet the relationship between maternal and calf vitamin A status is not well defined. This study evaluated cow–calf vitamin A status under restricted maternal vitamin A intake during late gestation and early lactation. Beef cows (*n* = 114) were individually fed 14,000 or 29,000 IU/d of vitamin A (approximately 30% or 75% of recommendations) from 128 days pre‐calving to 32 days post‐calving. All cows began with high liver retinol concentrations (mean ± SD: 786 ± 252 μg/g DM) from prior grazing. Cow liver and plasma, and calf liver and plasma, were sampled 32 ± 7 days postpartum. Cow liver retinol declined to 483 ± 182 μg/g DM by 32 days postpartum. Calf liver retinol averaged 51 ± 27 μg/g DM and plasma retinol 190 ± 47 ng/mL, both below adequacy thresholds. Calf plasma retinol increased with calf liver retinol (*p* < 0.01; *R* ^2^ = 0.22). Maternal post‐calving liver retinol explained 9.5% of the variation in calf liver retinol. These results indicate that adequate maternal liver vitamin A stores at calving do not ensure adequate calf vitamin A status when maternal dietary vitamin A is restricted.

## 1 Introduction

Vitamin A is well‐known for its role in vision, but it is also important for proper immune function and epithelial integrity, specifically in the gastrointestinal and respiratory tracts (Semba 1998). Clinical deficiency is rare in cow–calf systems, but marginal deficiencies can impact calf health and productivity. In fact, calves are generally the first to show signs of vitamin A deficiency because maternal requirements to maintain health and pregnancy are comparatively low (Guilbert and Hart 1935; Church et al. 1956). Calves are born with very low vitamin A stores, and their primary source at birth is colostrum (Stewart and McCallum 1938; Tomlinson et al. 1974). Vitamin A concentrations in colostrum have been reported to be 6 (Schweigert 1989) to 14 (Branstetter et al. 1973) times greater than that of milk, so colostrum is critical for establishing vitamin A stores in the young calf.

Vitamin A status can be assessed via plasma or liver samples. Plasma is easier to obtain but is a poor indicator of status because concentrations remain relatively constant unless liver stores are severely depleted or saturated (Blomhoff et al. 1990; Dowling and Wald 1958; Goodman 1984). In contrast, liver retinol, which contains ~90% of total body stores (McDowell 2000; Olson 1984), is more reflective of changes in dietary vitamin A intake, as it increases when vitamin A intake exceeds physiological need or decreases when vitamin A intake is insufficient to meet the body's needs (Olson 1984).

The objective of this study was to evaluate the relationship between cow and calf vitamin A status using plasma and liver samples under conditions of restricted maternal vitamin A intake during late gestation and early lactation. This scenario is relevant for beef cows transitioning from green fresh forage to stored feeds in the winter, where maternal liver stores may be high even as dietary vitamin A supply declines. Understanding whether maternal reserves alone are sufficient to ensure adequate calf vitamin A status has important implications for supplementation strategies of the gestating cow.

## 2 Materials and Methods

### 2.1 Cattle and Feeding

Research protocols were approved and monitored by the USDA, ARS, U.S. Meat Animal Research Center Institutional and Animal Care Committee in accordance with the Guide for the Care and Use of Agricultural Animals in Agricultural Research and Teaching (FASS 2010).

Composite breed (¼ Angus, ¼ Hereford, ¼ Simmental, ¼ Gelbvieh) cows in mid‐gestation (6.4 ± 1.2 years of age; *n* = 114) who had previously been grazing on pasture were utilized in this study. Initial BW of cows at the start of the study was 592 ± 58 kg and mean body condition score (BCS) on 1 to 9 scale was 5.6 ± 0.28. Cows received one of two different vitamin A supplementation levels: 9636 ± 464 IU/d (*n* = 29) or 24,923 ± 1692 IU/d vitamin A (*n* = 85) (Table 1). These levels were approximately 30% and 75% of the current NASEM (2016) recommendation of 2800 IU/kg dry matter (DM) for gestating beef cows weighing 592 kg consuming 2.0% of BW in DM per day (33,000 IU/d). They were individually fed using a Calan Broadbent Feeding system (Northwood, NH, USA) from 128 days pre‐calving to 32 days post‐calving. Cows were trained for 5 weeks prior to the start of the study to use the Calan gates. The ration consisted of pelleted supplement (0.41 ± 0.10 kg/d) that provided vitamin A in the form of retinyl acetate (Zhejiang NHU Company Ltd.; China) and the remainder consisted of 77% ground alfalfa/grass hay and 19% corn silage on a DM basis. Cows were fed once daily in the morning and orts were determined every 7 days. Mean vitamin A intake from the basal diet was 4393 ± 209 IU/d, which was estimated from the calculated basal dietary vitamin A concentration of 490 IU/kg. Basal dietary vitamin A was calculated assuming 1000 μg of β‐carotene is equal to 400 IU of vitamin A (McDowell 2000). Metabolic body size at BCS 5.5 was calculated as the cow's BW plus 45 kg for every BCS less than 5.5 or minus 45 kg for every BCS over 5.5, then raised to the 0.75 power (NRC 1996), and subsequent feed offered were calculated from this weight. Cows were fed to meet a ME maintenance requirement of 138 kcal ME/BW kg^0.75^. The amount of hay and corn silage was increased as pregnancy progressed to meet increased energy requirement of the conceptus (NASEM 2016). Cows maintained BCS during the trial with a mean BCS at 32 days post calving of 5.4 ± 0.31.

**TABLE 1:** Dietary vitamin A concentration of pellet and vitamin A intake (mean ± SD) for cows fed 30% or 75% of the NASEM (2016) vitamin A recommendation for gestating cows from 128 days pre‐calving to 32 days post‐calving.

	Supplementation level	
	30%	75%
Cows, n	29	85
Pellet vitamin A, IU/kg	23,612	61,048
Diet DMI, kg/d	9.39 ± 0.438	9.37 ± 0.452
	Vitamin A intake, IU/d	
Basal^a^	4401 ± 205	4391 ± 211
Pellet^b^	9636 ± 464	24,923 ± 1692
Total diet	14,037 ± 669	29,314 ± 1870

^a^ Basal vitamin A intake was calculated from forage and corn silage β‐carotene content assuming 1000 μg β‐carotene = 400 IU vitamin A (McDowell 2000).

^b^ Pellet provided 25 g Ca, 5 g Na, 102 mg Cu, 277 mg Zn, 148 mg Mn, 0.3 mg Se, 1.0 mg Co, 5.1 mg I, 2824 IU vitamin D, and 361 IU vitamin E per day.

### 2.2 Sample Collection and Analyses

Diet samples were collected daily and composited by week and were stored at −20°C. Diet samples were dried at 60°C for 72 h in a forced‐air oven to determine DM content and calculate DM intake. To determine dietary vitamin A concentration, freshly obtained basal diet samples (*n* = 4) and pellet samples (*n* = 2) were sent to the Michigan State University Veterinary Diagnostic Laboratory (Lansing, MI) for analysis. Basal diet samples were analyzed for β‐carotene and pellets for retinol concentration using ultraperformance liquid chromatography (UPLC) according to AOAC Official Method 2001.13 (AOAC 2011). Briefly, lipids were saponified with potassium hydroxide in ethanol, vitamins were extracted with hexane, dried under nitrogen, and reconstituted in mobile phase prior to chromatographic separation on a C18 column with photodiode array detection (450 nm for β‐carotene; 325 nm for retinol). Quantification was performed using six‐point external calibration curves prepared from certified standards. The limit of quantitation was 0.05 μg/g for β‐carotene and 20 ng/mL for retinol.

On Day 0 (128 days pre‐calving), cows were weighed and assessed to obtain a BCS. A blood sample (20 mL) and a liver biopsy were also collected at this time. Blood samples and liver biopsies were collected from cows again on Day 160 (32 days post‐calving), and from calves at 32 ± 7 days of age. All blood samples were collected via jugular venipuncture into EDTA tubes (BD Vacutainer; Becton, Dickinson, and Company, Franklin Lakes, NJ) and stored on ice and protected from light during transport to the lab. Within 2 h of collection, blood was centrifuged at 1500 × g for 20 min at 4°C. Plasma was immediately separated and transferred into amber microcentrifuge tubes, then stored at −80°C until analysis for vitamin A.

To obtain liver samples, cows were restrained in a hydraulic squeeze chute and calves were restrained standing in a calf chute. Liver biopsies were collected from the right side between the 11th and 12th ribs under local anesthesia using aseptic technique using the method of Engle and Spears (2000). Approximately 500 to 1000 mg of liver tissue was obtained with a modified Jamshidi bone marrow biopsy instrument (8‐gauge × 10 cm) and aspiration with a 20‐mL syringe. Liver samples were immediately rinsed with PBS and placed into 2‐mL amber microcentrifuge tubes, frozen in liquid N, and stored at −80°C until time of vitamin A analysis.

Liver samples were homogenized via pulverization using a cryogenic grinder (Freezer/Mill 6870D; SPEX Sample Prep, Metuchen, NJ) prior to vitamin A analysis. On the day of analysis, liver samples were retrieved from storage and immediately placed in liquid N. Tubes were then removed from liquid N and broken to remove tissue. Tissue was quickly placed in a weigh boat and placed on dry ice until transfer into cryogenic grinding vials resting in liquid N. A pre‐cooled stainless steel rod was placed in each vial, and vials were then capped with a room‐temperature stainless steel plug before placement into the cryogenic grinder. Once ground, liver samples were transferred from the vials into aluminum weigh boats sitting on dry ice. Samples were then transferred into 5‐ml microcentrifuge tubes and stored on dry ice until weighing. Pulverized samples were weighed into 50‐mL falcon tubes and placed on dry ice.

Vitamin A concentrations in liver and plasma samples were determined utilizing a portable fluorometer (iCheck Fluoro, BioAnalyt GmbH, Teltow, Germany). Extraction vials designed for use with the fluorometer contain 98% hexane, 1% isopropanol, and 1% ethanol. Distilled water was added to liver samples to achieve a dilution factor of approximately 100 or 50 for cow and calf liver samples, respectively. Falcon tubes were then capped and shaken by hand for 1 min. Immediately afterwards, 500 μL of solution was injected into extraction vials and shaken intensively for 10 s. Extraction vials were then centrifuged at 85 × *g* for 10 min to achieve phase separation. Vials were inserted into the fluorometer and vitamin A concentration was measured (excitation 340 nm, emission ≥ 400 nm). A pig liver was obtained and multiple subsamples were collected to test this extraction procedure prior to sample analysis. Accuracy of the procedure was verified by sending subsamples from this liver to a commercial laboratory (Michigan State University Veterinary Diagnostic Lab; Lansing, MI) where they were analyzed for vitamin A concentration via UPLC using modified procedures from Schmitz et al. (1991) and Rettenmaier and Schuep (1992). All readings were expressed as μg retinol equivalents per liter of liver. Once analyzed for vitamin A content, the remainder of the liver sample from each animal at each time point was dried for 24 h at 100°C in a forced‐air oven to determine DM. Vitamin A concentration of liver is reported as μg retinol/g DM.

Sample preparation of plasma was done as described by the iCheck Fluoro manufacturer. Briefly, plasma was thawed and 500 μL of plasma was injected into extraction vials and shaken intensively for 10 s. Vials were allowed to settle until phase separation was complete (~5 min). Vials were inserted into the fluorometer and retinol concentration was measured.

### 2.3 Statistical Analysis

Data were analyzed using SAS (SAS Institute Inc., Cary, NC) using PROC GLM. For all analyses, significance was declared at *p* ≤ 0.05 and tendencies at 0.05 < *p* ≤ 0.10. Model fit for all ANCOVA analyses was evaluated using the coefficient of determination (*R* ^2^), and the proportion of variation explained by each term was quantified by partial sums of squares.

#### 2.3.1 Cow Liver Retinol

To confirm that cows began the trial with comparable liver vitamin A stores prior to differential supplementation, initial (pre‐supplementation) liver retinol concentrations were compared between supplementation groups using a one‐way model with supplementation level as a fixed effect. To determine whether supplementation level influenced post‐calving liver stores after accounting for individual variation in initial liver retinol, post‐calving liver retinol concentrations were analyzed using an ANCOVA with supplementation level as a fixed effect and initial liver retinol as a covariate. No supplementation × liver interaction (*p* = 0.70) was detected and therefore the interaction term was removed.

#### 2.3.2 Cow Plasma Retinol

To test whether plasma retinol reflected liver retinol stores or supplementation level, cow plasma retinol at 32 days post‐calving was analyzed using ANCOVA including supplementation level as a fixed effect and cow post‐calving liver retinol as a covariate. No supplementation × liver interaction (*p* = 0.96) was detected and therefore the interaction term was removed. Because cow liver retinol was nonsignificant, a reduced model containing only supplementation level was also evaluated.

#### 2.3.3 Calf Liver–Plasma Relationship

The relationship between calf liver retinol and calf plasma retinol at 32 days of age was evaluated using ANCOVA, with calf plasma as the dependent variable, calf liver retinol as the covariate, and dam supplementation level as a fixed effect. No significant liver × supplementation interaction was detected (*p* = 0.84), and the interaction term was removed from the model.

#### 2.3.4 Maternal Diet and Liver Effects on Calf Liver and Plasma Retinol

To assess the extent to which maternal liver stores or dietary supplementation explained calf liver retinol, calf liver retinol was analyzed using ANCOVA with cow post‐calving liver retinol as a covariate and supplementation level as a fixed effect. No significant cow liver × diet interaction occurred (*p* = 0.13) and was therefore removed from the model. Calf plasma retinol was similarly analyzed using an ANCOVA containing cow liver, supplementation level, and their interaction. The interaction was not significant (*p* = 0.54) and was removed from the model.

## 3 Results and Discussion

### 3.1 Cow Liver Retinol

**FIGURE 1:**
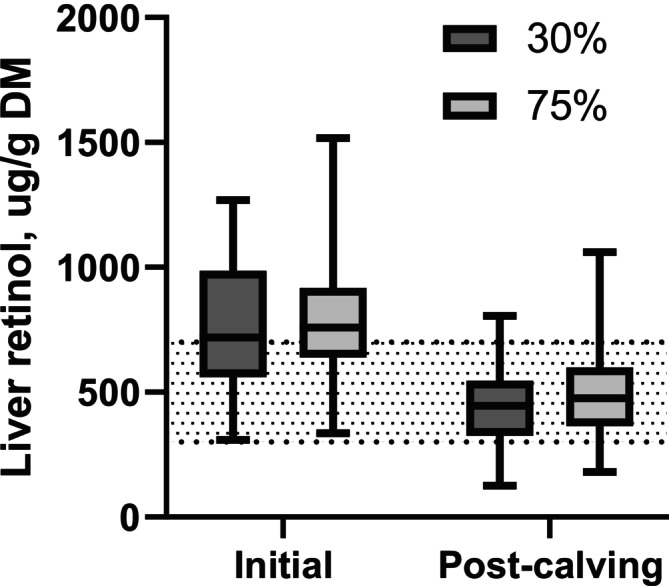
Liver retinol concentrations of cows receiving 30% (dark gray) or 75% (light gray) of recommended vitamin A supplementation during the final 128 days of gestation and first 32 days post‐calving, measured at study start (initial) and at 32 days post‐calving. Shaded area indicates adequacy range (300–700 μg/g DM; Puls 1994). Initial concentrations did not differ between supplementation groups (*p* = 0.76). Liver retinol declined in both groups over the study period (*p* < 0.01). However, after adjusting for initial concentrations, cows receiving 30% of the recommendation had lower post‐calving liver retinol than cows receiving 75% (*p* = 0.05). Box‐and‐whisker plots of the raw data are shown.

**FIGURE 2:**
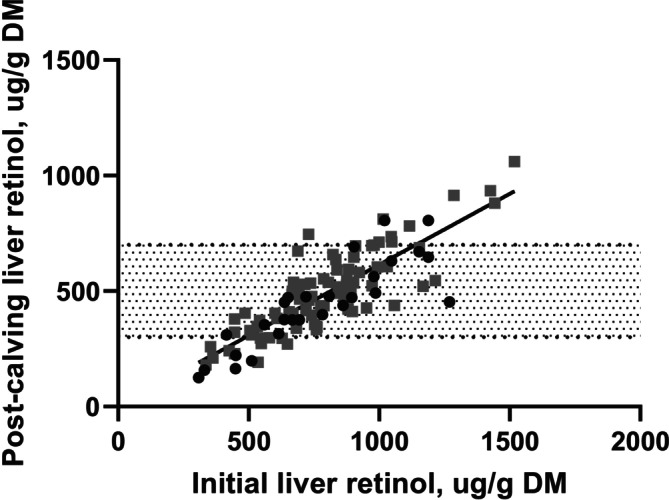
Relationship between initial (pre‐supplementation) and post‐calving liver retinol concentrations in beef cows fed either 30% (black circles) or 75% (gray squares) of the recommended vitamin A supplementation during the last 128 days of gestation and 32 days post‐calving. Shaded horizontal band represents the adequate reference range for liver retinol (300–700 μg/g DM; Puls 1994). Regression analysis (*R* ^2^ = 0.726, *p* < 0.01) indicated that initial liver retinol was a strong predictor (70.9%) of post‐calving liver retinol, whereas diet accounted for very little variation (1.1%), with no diet × initial liver interaction (*p* = 0.96).

Cows began the study with high liver retinol concentrations (mean 786 ± 252 μg/g DM; mean ± SD), reflecting recent grazing on green grass, and placing them at the high end of the adequate range (300–700 μg/g DM; Puls 1994). There was no difference (*p* = 0.76) in initial liver retinol concentrations among the two supplementation groups at 773 ± 50 and 790 ± 30 μg/g DM (mean ± SEM), for 30 and 75%, respectively (Figure 1). Over the subsequent 160 days, which included the final 128 days of gestation and 32 days post‐calving, cows received diets containing either 30% or 75% of the recommended supplemental vitamin A. At 32 days post‐calving, mean liver retinol concentration across supplementation groups had declined to 483 ± 182 μg/g DM (mean ± SD). An ANCOVA model including initial liver retinol concentration and supplementation treatment explained 72.6% of the variation in post‐calving liver retinol stores (*R* ^2^ = 0.726). Initial liver retinol concentration was a strong predictor (*p* < 0.01), accounting for 70.9% of the variation, whereas supplementation was also significant (*p* = 0.04) but accounted for only 1.1% (Figure 2). After adjusting for initial liver retinol concentration, cows receiving 30% of the recommended supplementation had lower (*p* = 0.05) post‐calving liver retinol concentrations than those fed 75% (Table 2). All cows experienced notable depletion of liver retinol during the study period. However, the majority of cows in both groups remained within the adequate range, with 82% and 87% of cows in the 30% and 75% supplementation groups, respectively, having post‐calving liver retinol concentrations above 300 μg/g of DM.

**TABLE 2:** Least squares means (± SEM) of retinol concentrations^a^ in cows and their calves when cows received 30% or 75% of recommended vitamin A supplementation during the final 128 days of gestation and first 32 days post‐calving.

	Cow supplementation level		*p*
	30%	75%	
Cow			
Initial liver retinol, μg/g DM	773 ± 50	790 ± 30	0.76
Post‐calving liver retinol, μg/g DM	449 ± 35	493 ± 20	0.05
Post‐calving plasma retinol, ng/mL	260 ± 7.9	275 ± 4.3	0.07
Calf			
Liver retinol, μg/g DM	42 ± 5.16	54 ± 2.89	0.06
Plasma retinol, ng/mL	169 ± 8.90	197 ± 4.92	< 0.01

*Note:* Cow measurements were obtained at study initiation (pre‐supplementation) and 32 days post‐calving; calf measurements were obtained at 32 days of age.

^a^ Adequacy thresholds (Puls 1994): cow liver = 300 μg/g DM; cow plasma = 300 ng/mL; calf liver = 100 μg/g DM; calf plasma = 225 ng/mL.

### 3.2 Cow Plasma Vitamin A

Plasma retinol concentrations 32 days post‐calving averaged 272 ± 40 ng/mL (mean ± SD), which falls below the adequate range of 300–800 ng/mL but above the marginal range of 100–200 ng/mL (Puls 1994). When cow plasma retinol was evaluated in a model including both supplementation level and cow post‐calving liver retinol concentration, cow liver retinol concentration (*p* = 0.24) was not significant. When cow post‐calving liver retinol concentration was removed from the model, there was a tendency (*p* = 0.07) for lower plasma retinol in cows fed 30% vs. 75% of the recommended supplementation rate (260 vs. 275; Table 2). Taken together, the intermediate plasma values likely reflect tight homeostatic regulation under restricted intake. The lack of a liver–plasma association is consistent with the regulation of circulating retinol, which changes little unless hepatic stores are severely depleted or saturated (Olson 1984; Frey et al. 1947). Under the feeding conditions in the current study, circulating retinol likely reflects hepatic homeostatic release with only a modest increment from dietary supply.

### 3.3 Calf Liver–Plasma Relationship

**FIGURE 3:**
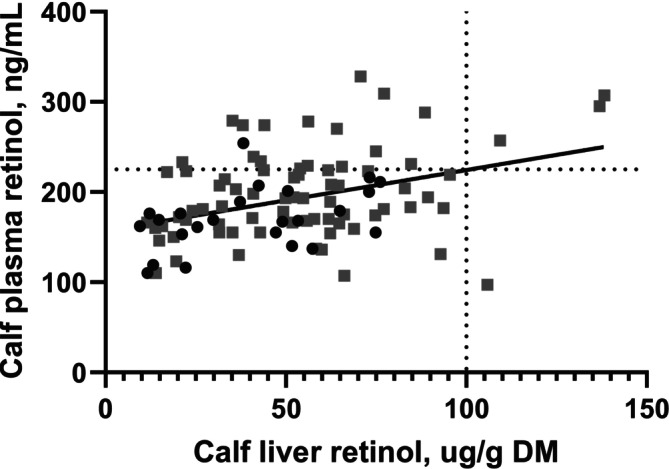
Relationship between calf liver retinol (μg/g DM) and plasma retinol (ng/mL) concentrations at 32 days of age. Each point represents an individual calf (*n* = 98). Dams were supplemented with either 30% (black circles) or 75% (gray squares) of the recommended vitamin A rate during the final 128 days of gestation and first 32 days post‐calving. Solid line represents the linear regression [*y* = 0.78 (± 0.163)*x* + 153.1 (± 9.30); *R* ^2^ = 0.19; *p* < 0.01]. Horizontal and vertical dotted lines indicate adequacy thresholds for plasma (225 ng/mL) and liver (100 μg/g DM) vitamin A, respectively (Puls 1994).

At 32 days of age, calf liver retinol concentration averaged 51 ± 27 μg/g DM (mean ± SD) and plasma retinol averaged 190 ± 47 ng/mL (mean ± SD), both below current adequacy thresholds (100–350 μg/g DM for liver; 225–325 ng/mL for plasma; Puls 1994). There was no significant interaction between calf liver retinol concentration and dam supplementation level (*p* = 0.84), indicating the slope of the relationship between calf liver and plasma did not differ between diet groups. The common slope across both supplementation levels was 0.78 ± 0.16 (SE) ng/mL plasma per μg/g DM liver (*p* < 0.01), with calf liver explaining 22.3% of the variation in plasma retinol (Figure 3). This relationship likely occurred because most calves (96%) had liver concentrations < 100 μg/g DM, a range where plasma retinol is more responsive to liver status. Swanson et al. (2000) also observed a positive relationship between liver and plasma vitamin A in calves at 28 days of age, with liver concentrations ranging from 10 to 110 μg/g wet basis (32 to 365 μg/g DM assuming 0.30% DM) and plasma concentrations from 66 to 114 ng/mL. While plasma increased with liver vitamin A in the Swanson et al. (2000) study, the slope of the relationship appeared to be relatively shallow at higher liver concentrations. This suggests that plasma is a more sensitive indicator when liver stores are low, but becomes less responsive as liver vitamin A approaches moderate levels. These findings reinforce the view that plasma vitamin A concentrations are tightly regulated and primarily reflect liver vitamin A status only when stores are low. Overall, these data suggest that calf liver vitamin A stores in the current study were not enough to sustain retinol concentrations in plasma and thus may not be adequate.

### 3.4 Maternal Diet and Liver Effects on Calf Status

When tested in an ANCOVA including cow liver retinol concentration post‐calving, supplementation level, the model explained approximately 8% of the total variation (*R* ^2^ = 0.079). Supplementation level significantly influenced calf plasma retinol concentration (*p* > 0.01), accounting for 6.6% of the variation, whereas cow liver retinol concentration post‐calving was not associated with calf plasma retinol (*p* = 0.39). Calves from 75% dams had 28 ng/mL greater plasma retinol concentration than calves from 30% dams (Table 2).

**FIGURE 4:**
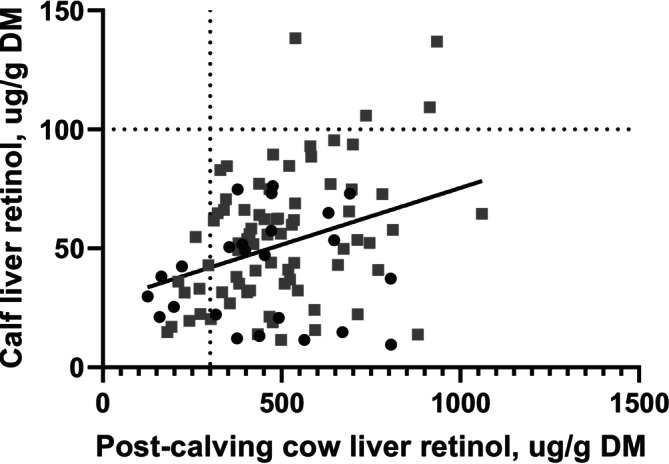
Relationship between calf liver retinol and post‐calving cow liver retinol for cows supplemented with either 30% (black circles) or 75% (gray squares) of the recommended vitamin A rate during the final 128 days of gestation and first 32 days post‐calving. Each point represents a cow–calf pair. Post‐calving cow liver retinol explained 9.5% of the total variation in calf liver retinol (*p* < 0.01), whereas supplementation level tended to be significant (*p* = 0.06). The regression line is fitted across both supplementation groups. Dashed horizontal and vertical lines indicate adequacy thresholds for calf (100 μg/g DM) and cow (300 μg/g DM) liver retinol concentrations, respectively (Puls 1994).

An ANCOVA model including cow liver retinol concentration post‐calving and cow vitamin A supplementation level explained 14.3% of the variation in calf liver retinol. Cow liver retinol post‐calving was significant (*p* < 0.01) after adjusting for diet (Figure 4), partial sums indicated that cow liver contributed 9.5% unique variation after adjusting for diet. Diet level tended to be (*p* = 0.06) significant, accounting for 3.2% of the variation, with calves from 75% dams exhibiting 11 μg/g greater liver retinol than calves from 30% dams (Table 2). Although cow liver retinol explained more variation than diet in this dataset, this may be due to the relatively low vitamin A intakes during late gestation and early post‐calving.

Branstetter et al. (1973) and Tomlinson et al. (1974) partitioned the vitamin A present in colostrum into contributions from dietary intake versus mobilization from maternal liver stores. In both studies, cows received alfalfa‐based diets that were described as providing a “high” intake of vitamin A, but the authors did not report measured β‐carotene concentrations of the forage. Therefore, total dietary vitamin A supply can only be estimated and should be interpreted cautiously. Based on typical β‐carotene concentrations for alfalfa hay and the supplemental vitamin A levels reported in those studies (45,000–50,000 IU/d), total vitamin A intake likely approached 75,000 to 100,000 IU/d. Under these high‐intake conditions, the authors found that approximately 40% of colostral vitamin A originated from mobilization of liver reserves, whereas roughly 60% was derived from vitamin A consumed in the diet during late gestation. In both studies, liver retinol concentrations of cows were maintained above 200 μg/g (wet basis; ~600 μg/g DM), which is well within the adequate range.

Cows in the present study received only 25%–40% of the dietary vitamin A estimated to be provided in the Branstetter et al. (1973) and Tomlinson et al. (1974) studies. Under these restricted intakes, a larger proportion of colostral vitamin A likely originated from mobilization of liver reserves, making maternal liver status appear more influential than it might be when cows consume higher levels of dietary vitamin A.

In the present study, most cows had liver retinol concentrations within or above (~85% ≥ 300 μg/g DM; Puls 1994), the commonly cited adequate range at the post‐calving sampling time, yet their calves had uniformly low liver retinol at 32 days of age (4% ≥ 100 μg/g DM). This indicates that maternal liver stores that are “adequate” by current reference thresholds are not sufficient, by themselves, to ensure adequate calf liver vitamin A when late‐gestation dietary vitamin A supply is restricted. In other words, cows can enter calving with apparently adequate hepatic reserves and still fail to transfer enough vitamin A to establish adequate calf stores.

These findings complement the results of Speer et al. (2024), who evaluated cows with initially low liver retinol concentrations (mean 186 μg/g DM) that were supplemented with 31,000, 93,000, or 155,000 IU/d from mid‐gestation through early lactation. Supplementing 93,000 IU/d or 155,000 IU/d of vitamin A increased cow liver retinol into the adequate range by calving and resulted in most calves (60 and 80%, respectively) achieving liver concentrations ≥ 100 μg/g DM at 32 days of age, whereas calves from cows fed the NASEM recommendation (31,000 IU/d) remained sub‐adequate. Because higher vitamin A intake in Speer et al. (2024) simultaneously increased both cow liver stores and the amount of vitamin A available directly in the diet, cow liver status and intake are confounded. Taken together with our data, however, the comparison suggests that simply having cow liver retinol within the traditional “adequate” range at calving is not enough to guarantee adequate calf stores when late‐gestation intake is low; rather, both sufficient maternal stores and dietary vitamin A supply in late gestation may be necessary to consistently achieve adequate calf liver retinol.

Overall, this study demonstrates that even when beef cows begin late gestation with adequate liver vitamin A reserves, restricted dietary vitamin A supply during late gestation and early lactation can result in sub‐adequate calf liver vitamin A concentrations at 1 month of age. Maternal liver stores alone were insufficient to maintain calf adequacy under low dietary supply, underscoring the importance of both maternal liver and dietary vitamin A during late gestation. These findings suggest that supplementation strategies for beef cows coming off high‐quality pasture should account for both current liver status and anticipated dietary vitamin A supply to ensure optimal vitamin A transfer to the calf.

## Funding

Funding for this study was provided by Great Plains Livestock Consulting Inc. (Eagle, NE) and the Nebraska Agricultural Experiment Station, with funding from the Hatch Multistate Research Program (NC 1181) from the USDA National Institute of Food and Agriculture. Mention of a trade name, proprietary product, or specific agreement does not constitute a guarantee or warranty by the USDA and does not imply approval of the inclusion of other products that may be suitable. USDA is an equal‐opportunity provider and employer.

## Conflicts of Interest

The authors declare no conflicts of interest.

## Data Availability

The data that support the findings of this study are available on request from the corresponding author. The data are not publicly available due to privacy or ethical restrictions.
